# Comparison of phenol-chloroform and a commercial deoxyribonucleic acid extraction kit for identification of bloodmeal sources from triatomines (Hemiptera: Reduviidae)

**DOI:** 10.1590/0037-8682-0189-2020

**Published:** 2020-11-25

**Authors:** Andressa Noronha Barbosa da Silva, Rita de Cássia Moreira de Souza, Nathan Ravi Medeiros Honorato, Rand Randall Martins, Antônia Claudia Jácome da Câmara, Lúcia Maria da Cunha Galvão, Egler Chiari

**Affiliations:** 1 Universidade Federal do Rio Grande do Norte, Centro de Ciências da Saúde, Programa de Pós-Graduação em Ciências Farmacêuticas, Natal, RN, Brasil.; 2 Universidade Federal de Minas Gerais, Instituto de Ciências Biológicas, Programa de Pós-Graduação em Parasitologia, Belo Horizonte, MG, Brasil.; 3 Fundação Oswaldo Cruz, Instituto René Rachou, Belo Horizonte, MG, Brasil.; 4 Universidade Federal do Rio Grande do Norte, Centro de Biociências, Programa de Pós-Graduação em Biologia Parasitária, Natal, RN, Brasil.; 5 Universidade Federal do Rio Grande do Norte, Centro de Ciências da Saúde, Programa de Pós-Graduação em Ciências Aplicadas à Saúde da Mulher, Natal, RN, Brasil.

**Keywords:** Triatominae, Gastrointestinal contentes, RNA ribosomal 12S, Polymerase Chain Reaction

## Abstract

**INTRODUCTION:**

Knowledge of triatomine bloodmeal sources is essential for understanding vector-host interactions in *Trypanosoma cruzi* transmission cycles. Expensive commercial deoxyribonucleic acid (DNA) extraction kits are widely used for bloodmeal identification. This study assessed the performance of an inexpensive phenol-chloroform DNA extraction protocol for identification of triatomine bloodmeal sources, comparing it with a commercially available kit.

**METHODS:**

Both methods were used to obtain DNA from the intestinal contents of *Triatoma brasiliensis* blood-fed on either *Columba* sp., *Mus musculus*, or *Gallus gallus*. Subsequently, the mitochondrial *12S* ribosomal ribonucleic acid (rRNA) gene was amplified by polymerase chain reaction, sequenced**,** and compared with GenBank data.

**RESULTS:**

Twelve (80%) samples extracted with the commercial kit and four (26.7%) with phenol-chloroform were pure (according to the A260/A280 ratio). Samples extracted with phenol-chloroform, except for *Columba* sp. samples, had higher DNA concentration than those extracted with the commercial kit. Samples extracted using phenol-chloroform and blood-fed on *G. gallus* had significantly higher DNA concentration than those blood-fed on *Columba* sp. (*p*-value <0.001) and *M. musculus* (*p*-value <0.001). The 215-base-pair *12S* rRNA fragment was amplified from all samples and produced reliable sequences, enabling the identification of the bloodmeal source, most of which showed identity and coverage above 95%. The phenol-chloroform method was much less expensive than the commercial kit but took considerably more time to perform.

**CONCLUSIONS:**

Our data showed that both DNA extraction methods produced reliable sequences enabling identification of triatomine bloodmeal sources but differed greatly in cost and time required.

## INTRODUCTION

Triatomines (Hemiptera, Reduviidae, and Triatominae) are obligatorily hematophagous insects in their nymphal and adult life cycle stages^1^. Therefore, they are potential transmitters of *Trypanosoma cruzi*, the etiological agent of Chagas disease or American trypanosomiasis^2^. Currently, between 6 and 7 million people are infected with *T. cruzi*, and more than 70 million live in areas at risk of infection^3^. The complex transmission cycle of this parasite includes two different kinds of hosts, a vertebrate and an invertebrate, and different developmental stages, and occurs in sylvatic, peridomestic, and domestic environments. The vertebrate hosts are numerous species of mammals from several orders, including Didelphimorphia, Rodentia, Carnivora, and Primates^4^. Despite their intrinsic refractoriness to *T. cruzi* infection^5^ ^,^ ^6^, birds and other cold-blooded animals impact the parasite transmission cycle and its investigation through coexistence in the same environment as reservoir host species, and are also a triatomine bloodmeals source^7^.

The vectorial capacity of triatomines is primarily determined by their association with human beings. Approximately ten triatomine species are of the greatest epidemiological importance due to their ability to colonize the domestic environment, where most vector-borne infections occur^8^ ^,^ ^9^. In Brazil, *Panstrongylus megistus* (Burmeister, 1835), *Triatoma brasiliensis* (Neiva, 1911), *Triatoma sordida* (Stål, 1859), *and Triatoma pseudomaculata* (Corrêa & Espínola, 1964) are epidemiologically important species in *T. cruzi* transmission because of their frequent invasion and colonization of human dwellings^10^.

Knowledge of the bloodmeal sources of triatomines is essential for understanding the vector-host interactions involved in *T. cruzi* transmission cycles^11^. The first tools used to determine the bloodmeal sources of these insects were based on immunological tests: the precipitin reaction^12^, the complement fixation test (based on detection of host antibodies)^13^, and enzyme-linked immunosorbent assays (ELISA)^14^. However, the success of these techniques is limited by the amount of blood ingested by the insects, the degradation of bloodmeal contents following digestion within the gut of the insect, and an inability to determine the bloodmeal source to species-level. After the advent of molecular biology, more recent studies have demonstrated that a combination of polymerase chain reaction (PCR)^15^, deoxyribonucleic acid (DNA) cloning, and DNA sequencing of the *cytochrome* c *oxidase I* (*coi*) and *cytochrome b* (*cytb*) genes^16^can be used for bloodmeal identification of triatomines. A variety of different DNA extraction methods are available, including commercial kits, each with advantages and disadvantages^17^. DNA-sequence-based methods of bloodmeal identification require high-quality purified DNA samples, which may necessitate the use of expensive DNA extraction procedures. In an attempt to optimize the cost of bloodmeal identification, we aimed to assess the performance of an inexpensive phenol-chloroform DNA extraction protocol for identification of sources of triatomine bloodmeals, comparing it with that of a commercial DNA extraction kit.

## METHODS

### Experimental groups

Thirty *T. brasiliensis* first-stage nymphs from the insectary of Laboratório de Referência de Triatomíneos e Epidemiologia da Doença de Chagas (LATEC/IRR/FIOCRUZ/MINAS) were randomly divided into three groups of 10 individuals after hatching. The use of first-stage nymphs ensured control over the type of bloodmeal that was offered to each group. After ten days of fasting, the insects were exposed to one of three different bloodmeal sources: *Columba livia* (domestic pigeon, group 1), *Mus musculus* (mouse, group 2)*,* and *Gallus* (chicken, group 3). The vertebrate hosts were anesthetized following a protocol (LM 10/18) approved by the Animal Use Ethics Committee of FIOCRUZ (CEUA/FIOCRUZ). Domestic pigeons were anesthetized via the pectoral intramuscular route with detomidine (0.05 mg/kg) + ketamine (10-25 mg/kg), mice were anesthetized via the intraperitoneal route with ketamine (100-200 mg/kg) + xylazine (5-16 mg/kg), and chickens were anesthetized via the pectoral intramuscular route with detomidine (0.3 mg/kg) + ketamine (20 mg/kg).

The nymphs were weighed before (variation between 0.08 mg and 0.11 mg) and after (variation between 0.30 mg and 0.45 mg) blood-feeding. Regardless of the amount of blood ingested, all insects were dissected and their bloodmeal sources were analyzed.

### DNA extraction

Two days after the bloodmeal, the nymphs were dissected using tweezers and scissors, and their midgut and hindgut were removed and stored in a 1.5 mL tube. DNA from five individuals from each group was extracted directly from the intestinal content using the DNeasy Blood & Tissue Kit (Qiagen, Hilden, Germany) according to the manufacturer’s instructions. The remaining five individuals from each group were added to 400 μL of 0.2 M Guanidine-HCl 6 M/EDTA (Invitrogen, Carlsbad, CA, USA). The DNA was then extracted using the phenol-chloroform method, according to Gomes et al.^18^, with some modifications. Three hundred microliters (compared to the 200 μL recommended in the original protocol) of each diluted sample was transferred to a new tube, and 100 μL of phenol Tris pH 8.0 (Invitrogen™ UltraPure™ Phenol) and 100 μL of chloroform (Merck, Darmstadt, Germany) were added. The mixture was slowly homogenized for 2 min and centrifuged at 3,400 × *g* for 5 min. Next, the supernatant was transferred to another tube, and 200 μL of ultrapure water was added to the remaining sediment. Once again, the new mixture was slowly homogenized for 2 min and centrifuged at 3,400 × *g*. The supernatant was transferred to a fresh tube, and the sediment was discarded. Next, 320 μL of chloroform was added to the supernatant, and the mixture was slowly homogenized for 2 min and centrifuged at 3,400 × *g*. In order to obtain the maximum quantity of DNA, we transferred approximately 300 μL (instead of the 240 μL recommended) of the supernatant to a new tube containing 100 mM of sodium acetate (Merck), 40 μg of glycogen (Invitrogen), and two volumes of absolute ethanol (Merck), and then incubated this mixture in an ice bath for 15 min to precipitate the DNA. After that, the samples were centrifuged at 15,900 × *g* for 15 min, and the supernatant was discarded. Finally, after the volatilization of the ethanol, the DNA was resuspended with 30 μL (instead of the 20 μL indicated in the previous protocol) of ultrapure sterile water and stored at -70 °C.

DNA concentration and quality were determined using a Nanodrop 2000 Spectrophotometer (Thermo Scientific, Waltham, MA, USA). The absorbance ratio at 260 nm and 280 nm (A260/A280 ratio) and the absorbance ratio at 260 nm and 230 nm (A260/A230 ratio) were used to assess protein and phenol contamination, with higher values associated with better DNA quantity and purity. DNA with an A260/A280 ratio between 1.8 and 2.0, and an A260/A230 ratio between 2.0 and 2.2, was considered pure^19^. Afterwards, 5 ng of each sample was analyzed by electrophoresis on a 1% agarose gel and visualized using GelRedTM (100×) to determine the integrity of the extracted DNA (Supplementary Figure 1).

###  PCR of the mitochondrial *12S* ribosomal ribonucleic acid (rRNA) gene 

PCR reactions were performed using 10 ng of template DNA in a final reaction volume of 25 μL consisting of 2.5 μL of 10× buffer, 2.5 μL of 2.5 mM dNTPs, 0.75 μL of 50 mM MgCl_2_, 25 pmol of each primer, and 0.125 U of *Taq* Platinum (Invitrogen). The primers used were the forward L1085 (5’-CCCAAACTGGGATTAGATACCC-3’) and the reverse H1259 (5’- GTTTGCTGAAGATGGCGGTA-3’), which amplify a 215 base pairs (bp) fragment. These primers were previously designed to bind conserved regions of the *12S* rRNA locus^20^. Both negative (no DNA) and positive (DNA of the intestinal contents of a triatomine fed on *G. gallus*) controls were included in each PCR assay.

PCR was conducted for 35 cycles of denaturation at 95 °C for 30 s, annealing at 57 °C for 15 s, and extension at 72 ºC for 30 s, using a Veriti™ Thermal Cycler (Applied Biosystems, Foster City, CA, USA). Three microliters of each PCR product were analyzed by electrophoresis on a 1% agarose gel and visualized using GelRedTM (100×).

Subsequently, PCR products were purified using the QIAquick PCR Purification Kit (Qiagen), following manufacturer’s instructions. Then, the concentration of DNA was measured using a NanoDrop 2000 Spectrophotometer (Thermo Scientific) to adjust it for the sequencing reaction.

### Sequencing and analysis

The purified PCR products were directly sequenced using the BigDye® Terminator v 3.1 Cycle Sequencing Kit (Applied Biosystems). For these reactions, the following were used: 10 ng of purified DNA, 5 pmol of the forward (L1085) and reverse (H1259) primers, 1.75 μL of sequencing buffer (200 mM Tris-HCl, pH 9; 5 mM MgCl_2_), 0.5 μL of BigDye, and water up to 10 μL. One sequencing reaction was performed for each primer. The products were precipitated and processed on an automatic sequencer ABI 3730XL (Applied Biosystems) on the platform at the René Rachou Institute/FIOCRUZ/Minas. The electropherograms of the obtained sequences were evaluated using BioEdit Sequence Alignment Editor® V. 7.0.9.0^21^. To identify the sources of triatomine bloodmeals, the resulting sequences were compared with sequences deposited in GenBank using Basic Local Alignment Search Tool for nucleotide (BLASTn) (http://www.ncbi.nlm.nih.gov/). The same experimental procedures (i.e., PCR conditions, DNA purification, and DNA sequencing) were used for samples obtained by both DNA extraction methods.

### Comparison of costs and time between extraction methods

The estimated costs of the commercial kit and each reagent used in the extraction with phenol-chloroform were checked on the respective websites of the manufacturing companies. The cost-benefit ratio was calculated by dividing the price of each reagent by the number of possible reactions per bottle. For this comparison, the acquisition and maintenance costs of the centrifuge and pipettes as well as the tips and water used in the procedures, were not considered. The time spent in the execution of each method was also calculated, without considering the handling differences between the procedures, but labor costs were not considered.

### Statistical analysis

The data were initially analyzed using the Kolmogorov-Smirnov test to verify distribution normality, and either parametric or non-parametric statistical tests were subsequently performed depending on the normality of the data. Student’s *t*-test (parametric data) or Mann-Whitney *U* test (non-parametric data) were used to compare the average DNA concentrations extracted from the intestinal contents of triatomines as well as the A260/A280 and A260/A230 ratios. For comparisons of the average DNA concentrations of individuals blood-fed on the three different bloodmeal sources, either one-way analysis of variance (ANOVA) followed by the Bonferroni post-hoc test (parametric data) or the Kruskal-Wallis *H* test (non-parametric data) were used^22^. The significance level was set at a two-tailed 5% level. Stata 15 (Stata Corporation, College Station, TX, USA) was used for statistical analysis.

## RESULTS

We determined and compared the reliability of bloodmeal identification when using DNA extracted either with a phenol-chloroform method or a commercial DNA extraction kit. The A260/A280 ratios and DNA concentrations are shown in Table 1. Of the 15 samples extracted using the commercial kit, 12 (80%) had an A260/280 ratio between 1.8 and 2.0 and were regarded as being “pure samples”. In contrast, only four samples (26.7%) obtained using the phenol-chloroform method had the same quality level (Table 1). There was no significant difference in the average A260/280 ratios of DNA samples collected from triatomines fed on the same bloodmeal source but extracted using different methods: (**1**) for *Columba* sp., the mean and standard deviation for the commercial kit were 1.84 ± 0.02*,* and for phenol-chloroform were 1.78 ± 0.08 (*p*-value = 0.916); (**2**) for *M. musculus*, the average for the commercial kit was 1.76 ± 0.12*,* and for phenol-chloroform was 1.67 ± 0.16 (*p*-value = 0.892); and (**3**) for *G. gallus*, the average for the commercial kit was 1.83 ± 0.01, and for phenol-chloroform was 1.75 ± 0.04 (*p*-value = 0.995). With regard to the A260/A230 ratio, although for both methods the presence of some contamination was apparent, the samples extracted with the commercial kit showed values closer to the appropriate range when compared to those extracted using phenol-chloroform. Analyzing the average values we noticed that (**1**) for *Columba* sp*.*, the mean and standard deviation for the commercial kit were 1.40 ± 0.21 and for phenol-chloroform were 0.08 ± 0.01 (*p*-value <0.001); (**2**) for *M. musculus*, the average for the commercial kit was 0.34 ± 0.02 and for phenol-chloroform was 0.21 ± 0.2 (*p*-value = 0.210); and (**3**) for *G. gallus*, the average for the commercial kit was 1.27 ± 0.29 and for phenol-chloroform was 0.3 ± 0.0 (*p*-value = 0.001).

**TABLE 1:** Bloodmeal source, DNA concentration, and A260/A280 and A260/A230 ratios of samples extracted using either a commercial kit or the phenol-chloroform method.

Type of extraction / bloodmeal supplied	Sample	DNA concentration (ng/µL)	A260/280	A260/A230
**Commercial kit**				
*Columba* sp. (Domestic pigeon)	1	40.46	1.81	1.27
	2	41.70	1.88	1.23
	3	92.21	1.84	1.76
	4	51.59	1.84	1.38
	5	44.85	1.84	1.35
*Mus musculus* (Mouse)	6	5.27	1.58	0.32
	7	5.86	1.78	0.32
	8	7.08	1.70	0.38
	9	6.78	1.84	0.34
	10	6.81	1.88	0.34
*Gallus gallus* (Chicken)	11	50.37	1.84	1.39
	12	51.31	1.83	1.41
	13	28.43	1.83	0.76
	14	61.89	1.83	1.39
	15	48.45	1.84	1.41
**Phenol-chloroform**				
*Columba* sp. (Domestic pigeon)	1	17.91	1.75	0.07
	2	17.87	1.72	0.09
	3	26.31	1.80	0.10
	4	14.59	1.91	0.07
	5	15.75	1.72	0.07
*Mus musculus* (Mouse)	6	22.54	1.69	0.13
	7	30.21	1.91	0.13
	8	20.75	1.57	0.10
	9	41.85	1.69	0.12
	10	29.90	1.47	0.56
*Gallus gallus* (Chicken)	11	60.81	1.73	0.21
	12	81.74	1.70	0.30
	13	82.11	1.79	0.28
	14	109.43	1.72	0.37
	15	94.66	1.80	0.32

**A260/A280:** ratio of the absorbance at 260 nm and 280 nm; **A260/A230:** ratio of the absorbance at 260 nm and 230 nm; **DNA:** deoxyribonucleic acid.

**FIGURE 1:**
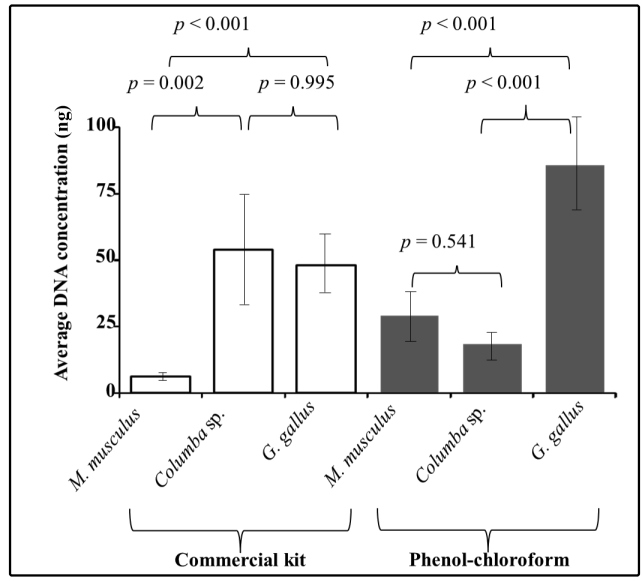
Comparison of the average deoxyribonucleic acid (DNA) concentration obtained when using different DNA extraction methods on samples from individuals fed on different bloodmeal sources. Samples were compared using analysis of variance (ANOVA), followed by post-hoc Bonferroni test: *p* <0.05. Graphs represent the mean ± standard deviation of the DNA concentrations in nanograms/microliters.

For the commercial kit, the DNA concentration ranged from 5.27 ng/µL to 92.21 ng/µL, with the lowest DNA concentration recorded for an individual blood-fed on *M. musculus* and the highest on *Columba* sp. For the phenol-chloroform method, the DNA concentration varied from 14.59 ng/µL for individuals blood-fed on *Columba* sp*.* to 109.43 ng/µL for those fed on *G. gallus* (Table 1). The average DNA concentrations of samples derived from different bloodmeal sources, but extracted using the same method, were also compared. For samples extracted with the commercial kit, the average DNA concentration of *M. musculus* samples was significantly lower than those observed for *Columba* sp. and *G. gallus*, with *p*-values lower than 0.002 and 0.001, respectively. For extraction with phenol-chloroform, the average DNA concentration of samples derived from *G*. *gallus* was significantly higher than those of either *Columba* sp. (*p*-value <0.001) or *M. musculus* (*p*-value <0.001) (Figure 1).

Table 2 shows the average and standard deviation of the DNA concentration of samples from different bloodmeal sources according to the extraction method. The highest DNA yields were observed for samples derived from *G. gallus* and were achieved with the phenol-chloroform method: mean of 85.75 ± 17.99 ng/μL. In contrast, samples derived from *Columba* sp. and extracted using the commercial kit were significantly more concentrated than those extracted using the phenol-chloroform method (*p*-value = 0.02). The integrity of the extracted DNA using either the commercial kit or phenol-chloroform was evaluated by agarose gel electrophoresis. We observed that a larger number of samples extracted using phenol-chloroform appeared to be degraded (Supplementary Figure 1). Despite the apparent degradation of some samples, it was still possible to successfully amplify and visualize on agarose gels the PCR-amplified 215 bp fragment of the *12S* rRNA gene for all samples (Supplementary Figure 2). All samples extracted with the commercial kit (*n* = 15) and with phenol-chloroform (*n* = 15) produced reliable DNA sequences enabling identification of the vertebrate host species used for the bloodmeal. A total of 15 samples (eight extracted with the commercial kit and seven with phenol-chloroform) presented similar sequences to those deposited in NCBI, with 100% identity and 100% coverage by BLAST. Only two samples extracted by the commercial kit, collected from triatomines fed on *Columba* sp. and *M. musculus*, had identities lower than 90% (84% and 88%, respectively). Max scores and E-values suggested that the sequences obtained from samples extracted using both methods are sufficiently reliable for molecular applications (Table 3). 

**TABLE 2:** Comparison of the average DNA concentration obtained with a commercial kit or a phenol-chloroform DNA extraction method.

Food source	Extraction method		***p*-value**
	Commercial kit	Phenol-chloroform	
	ng/μL ± SD	ng/μL ± SD	
*Columba* sp*.*	54.16 ± 21.70	18.49 ± 4.60	0.020
*Mus musculus*	6.36 ± 0.76	29.05 ± 8.32	0.004
*Gallus gallus*	48.09 ± 12.17	85.75 ± 17.99	0.006

**DNA:** deoxyribonucleic acid; **SD:** standard deviation Averages compared using the Student's t-test (*p* <0.05) after checking for normality (Kolmogorov-Smirnov test).

**TABLE 3:** BLAST results of the top hits for the mitochondrial *12S* rRNA sequences derived from DNA samples obtained using a commercial kit or a phenol-chloroform extraction method.

Type of extraction / bloodmeal supplied	Sample	Max Score/	Query Coverage	E	Identity	Organism	GenBank
		Total Score	(%)	value	(%)		code
**Commercial kit**							
*Columba* sp. (Domestic pigeon)	1	237/237	100	9e-59	99	*Columba* sp.	KX902246.1
	2	172/172	100	3e-39	90	*Columba* sp.	KX902246.1
	3	237/237	97	9e-59	100	*Columba* sp.	KF926376.1
	4	117/117	93	1e-22	84	*Columba* sp.	KX902246.1
	5	204/204	97	2e-48	96	*Columba* sp.	KX902246.1
*Mus musculus* (Mouse)	6	224/224	100	5e-55	98	*M. musculus*	KX381752.1
	7	150/150	100	1e-32	88	*M. musculus*	KR020499.1
	8	237/237	100	9e-59	100	*M. musculus*	KX381752.1
	9	243/243	100	2e-60	100	*M. musculus*	KP168712.1
	10	243/243	100	2e-60	100	*M. musculus*	KP168712.1
*Gallus gallus* (Chicken)	11	243/243	100	2e-60	100	*G. gallus*	KX987152.1
	12	243/243	100	2e-60	100	*G. gallus*	KX987152.1
	13	237/237	100	9e-59	100	*G. gallus*	KX987152.1
	14	237/237	100	9e-59	100	*G. gallus*	KX987152.1
	15	237/237	100	9e-59	100	*G. gallus*	KX987152.1
**Phenol-chloroform**							
*Columba* sp. (Domestic pigeon)	1	218/218	99	2e-53	98	*Columba* sp.	KX902246.1
	2	224/224	100	2e-54	98	*Columba* sp.	KF926376.1
	3	241/241	99	7e-60	100	*Columba* sp.	KX902246.1
	4	243/243	100	2e-60	100	*Columba* sp.	KX902246.1
	5	224/224	100	2e-54	98	*Columba* sp.	KF926376.1
*Mus musculus* (Mouse)	6	237/237	100	9e-59	100	*M. musculus*	KX381752.1
	7	237/237	100	9e-59	100	*M. musculus*	KX381752.1
	8	237/237	100	9e-59	100	*M. musculus*	KX381752.1
	9	228/228	100	4e-56	98	*M. musculus*	KX381752.1
	10	223/223	99	2e-57	98	*M. musculus*	KX381752.1
*Gallus gallus* (Chicken)	11	243/243	100	2e-60	100	*G. gallus*	AJ849444.2
	12	200/200	90	1e-47	97	*G. gallus*	KX987152.1
	13	237/237	100	9e-59	100	*G. gallus*	KX987152.1
	14	237/237	100	9e-59	100	*G. gallus*	KX987152.1
	15	202/202	100	6e-48	95	*G. gallus*	KX987152.1

**BLAST:** Basic Local Alignment Search Tool; **rRNA:** ribosomal ribonucleic acid; **DNA:** deoxyribonucleic acid.

The cost-benefit analysis showed that the extraction of DNA by the phenol-chloroform method was approximately 36 times less expensive than the commercial kit. The commercial kit was the fastest method to perform and could be completed in 30 min compared to 105 min using the phenol-chloroform protocol (Table 4).

**TABLE 4:** Comparison of the estimated cost per sample using either a commercial DNA extraction kit or a phenol-chloroform method.

Extraction method/reagent	Quantity of reagent	Reagent cost	Number of reactions	Estimated cost per	Assay time
	per bottle	(US$)	per bottle	Sample (US$)	(min)
**Commercial kit**					**30**
DNeasy Blood & Tissue Kit	-	1,350.00	250	5.4	
**Phenol-chloroform**					**105**
Phenol	400 mL	290.00	4,000	0.07	
Chloroform	1000 mL	70.00	2,500	0.03	
Ethanol	1000 mL	90.00	1,724	0.05	
Glycogen	10 g	325.00	125,000	<0.01	
Sodium acetate	1000 g	90.00	461,538	<0.01	
Guanidine	100 g	81.00	2,625	<0.01	
EDTA	100 g	40.00	33,500	<0.01	
Overall cost	-	986.00	-	0.15	

**EDTA:** Ethylenediamine tetraacetic acid; **DNA:** deoxyribonucleic acid.

## DISCUSSION

The epidemiological scenario of Chagas disease has become more complex because of anthropogenic changes to natural environments caused by deforestation, and the subsequent occupation of such areas by human beings. Thus, the classic separation of the transmission cycles of *T. cruzi* in either sylvatic or domestic settings may either not exist or vary in different places^23^. In this context, the study of the sources of triatomine bloodmeals is of fundamental importance for understanding the dynamics of parasite transmission in different regions, thus supporting epidemiological surveillance activities and helping to prevent the infection of humans and domestic animals. 

In this study, the performance of an existing protocol for the extraction of DNA by the phenol-chloroform method was assessed for identification of triatomine bloodmeal sources. Our data show that the concentration and purity of the DNA extracted with this method were similar to those obtained using a commercial kit. For instance, DNA extracted from triatomine intestinal contents, using the phenol-chloroform method, was successfully amplified by conventional PCR. This suggests that the quality of DNA extracted using this method is similar to that of the commercial kit, at least for this purpose, in accordance with a previous report^24^. Additionally, it was possible to accurately identify the bloodmeal sources of all the analyzed samples, with BLAST sequence identities greater than 95%, when compared to the GenBank database. Moreover, the BLAST E-values were close to zero, suggesting that the phenol-chloroform method is capable of extracting DNA with enough quality to generate sufficiently reliable and biologically accurate results for host species identification^25^ ^,^ ^26^. Therefore, we infer that both DNA extraction methods are equally efficient for bloodmeal identification of Chagas disease vectors under laboratory conditions.

In several previous studies, the extraction of total DNA for the identification of the source of triatomine bloodmeals was usually performed using the DNeasy Tissue Kit (Qiagen)^7^ ^,^ ^24^, and identification of the bloodmeal source was possible for most of the tested samples. Our data demonstrated that the commercial kit is efficient in detecting the bloodmeal sources of Chagas disease vectors. However, the use of a commercial kit makes the identification process more expensive, owing to the greater cost of the product. 

Penã et al.^23^ also used the phenol-chloroform method for DNA extraction to identify the sources of triatomine bloodmeals. These authors used a modified version of the protocol described by Sambrook, Fritsch, and Maniatis^27^. They then performed a high resolution melting real-time PCR analysis targeting the *cytb* gene, and sequenced the DNA samples to identify the bloodmeal source, thus validating this DNA extraction method. In contrast, we performed DNA extraction using the protocol described by Gomes et al.^18^. We used these samples in conventional PCR targeting the mitochondrial *12S* rRNA gene, which generated products that could be successfully sequenced. Our results, which were obtained using insects blood-fed under laboratory conditions, were similar to those of Penã et al.^23^, showing that phenol-chloroform extraction is efficient for obtaining total DNA in this context. DNA extraction by the phenol-chloroform method is widely used for the identification of microorganisms, such as viruses, bacteria, and protozoa, from various types of samples (blood, serum, tissue, and cerebrospinal fluid)^28^ ^-^ ^31^. Corroborating other studies^32^, this is a methodology that, when compared with others, has a similar effectiveness and a better cost-benefit. However, careful handling of the reagents both before and after the application of the technique is essential, as both phenol and chloroform can cause environmental contamination and have toxic effects (hepatotoxicity, nephrotoxicity, and carcinogenesis) for humans and other animals^33^ ^,^ ^34^.

The blood-feeding habits of triatomines are variable^35^. Some species are more often associated with birds, such as *T. pseudomaculata* and *T. sordida*, while others show an apparent preference for mammals, for example, *P. megistus*, *Triatoma rubrofasciata* (De Geer, 1773)*,* and *T. brasiliensis* ^1^. *Triatoma brasiliensis*, the species of triatomine used in this study, has great eclecticism regarding the bloodmeal source, and DNA from animals of different orders of mammals, birds, and reptiles can be detected in the intestinal contents of this triatomine^7^ ^,^ ^36^. In this study, when using phenol-chloroform extraction, it was possible to satisfactorily identify, with similar success, the presence of DNA from animals of two orders of birds and one of mammals.

It is important to state clearly that, in our study, both methods exhibited deficiencies, causing high levels of contamination of the samples, as demonstrated by the A260/A230 ratio. Dilhari et al. (2017)^37^ also demonstrated higher levels of this ratio using the same commercial kit compared to the phenol-chloroform method. This may be related to the fact that the quantity and purity of DNA extracted with the phenol-chloroform method can be underestimated due to the high extinction coefficient of phenol at 260 nm^38^. Furthermore, the numerous steps and manipulations of tubes required during the phenol-chloroform method may also influence the level of contamination. High levels of phenol, peptides, and/or carbohydrates can affect the performance of PCR reactions^27^ ^,^ ^39^ ^,^ ^40^ and, consequently, increase the difficulty of identifying bloodmeal sources. Even so, the phenol-chloroform DNA extraction technique has proved to be functional. Samples extracted using this method could be amplified by PCR to identify the source of triatomine bloodmeal. The significantly lower cost, and results comparable to those obtained using the commercial kit, suggest the great potential of the phenol-chloroform method as a tool for investigating the interaction of triatomines with different vertebrate hosts of *T. cruzi*.
