# Supplementary figures and images for: Comparison of phenol-chloroform and a commercial deoxyribonucleic acid extraction kit for identification of bloodmeal sources from triatomines (Hemiptera: Reduviidae)
Source: Rev Soc Bras Med Trop. 2020 Nov 25;53:e20200189. doi: 10.1590/0037-8682-0189-2020 (PMC7723369; doi:10.1590/0037-8682-0189-2020)

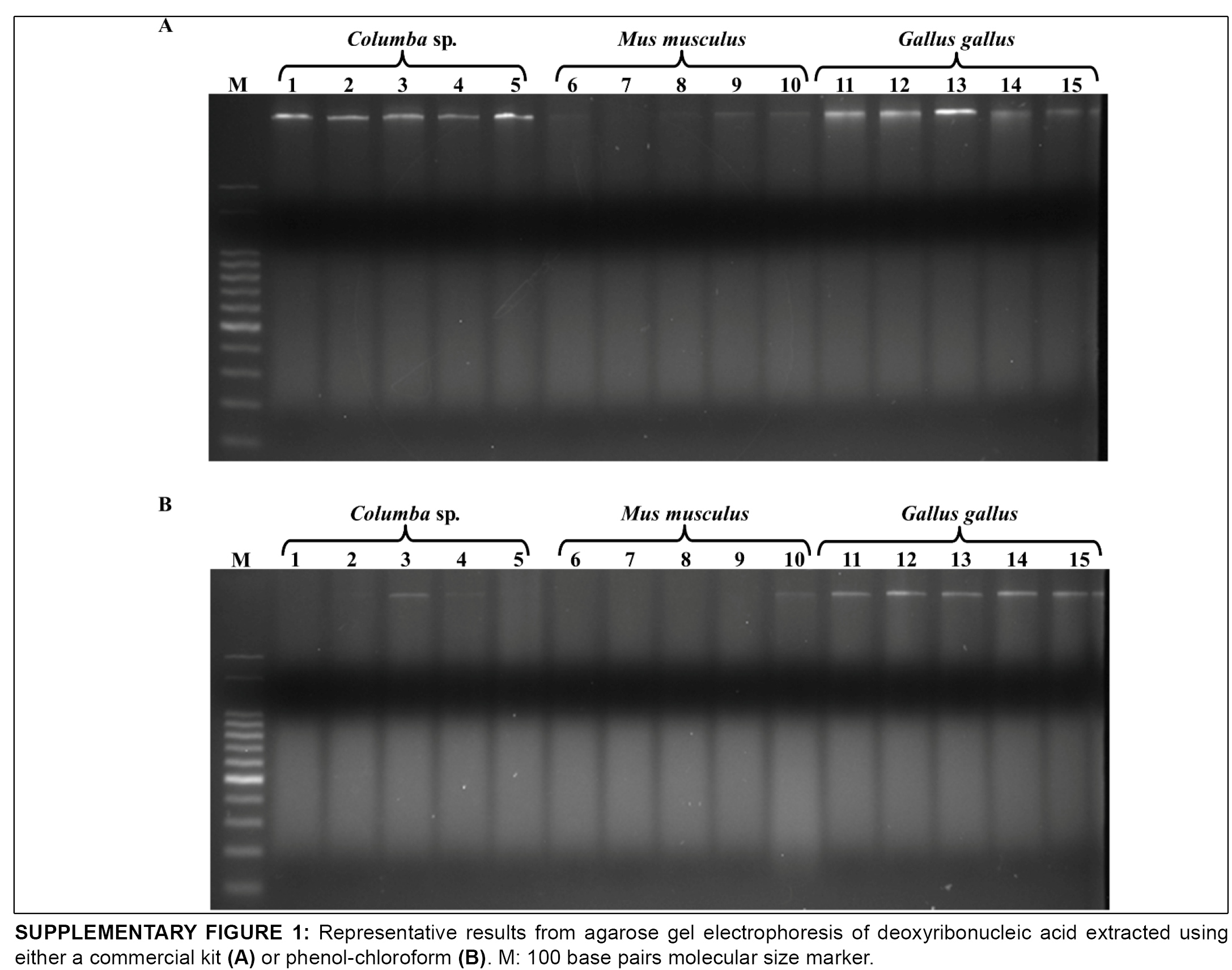

Supplement: Supplementary file 1 [file 1678-9849-rsbmt-53-e20200189-suppl1.jpg]

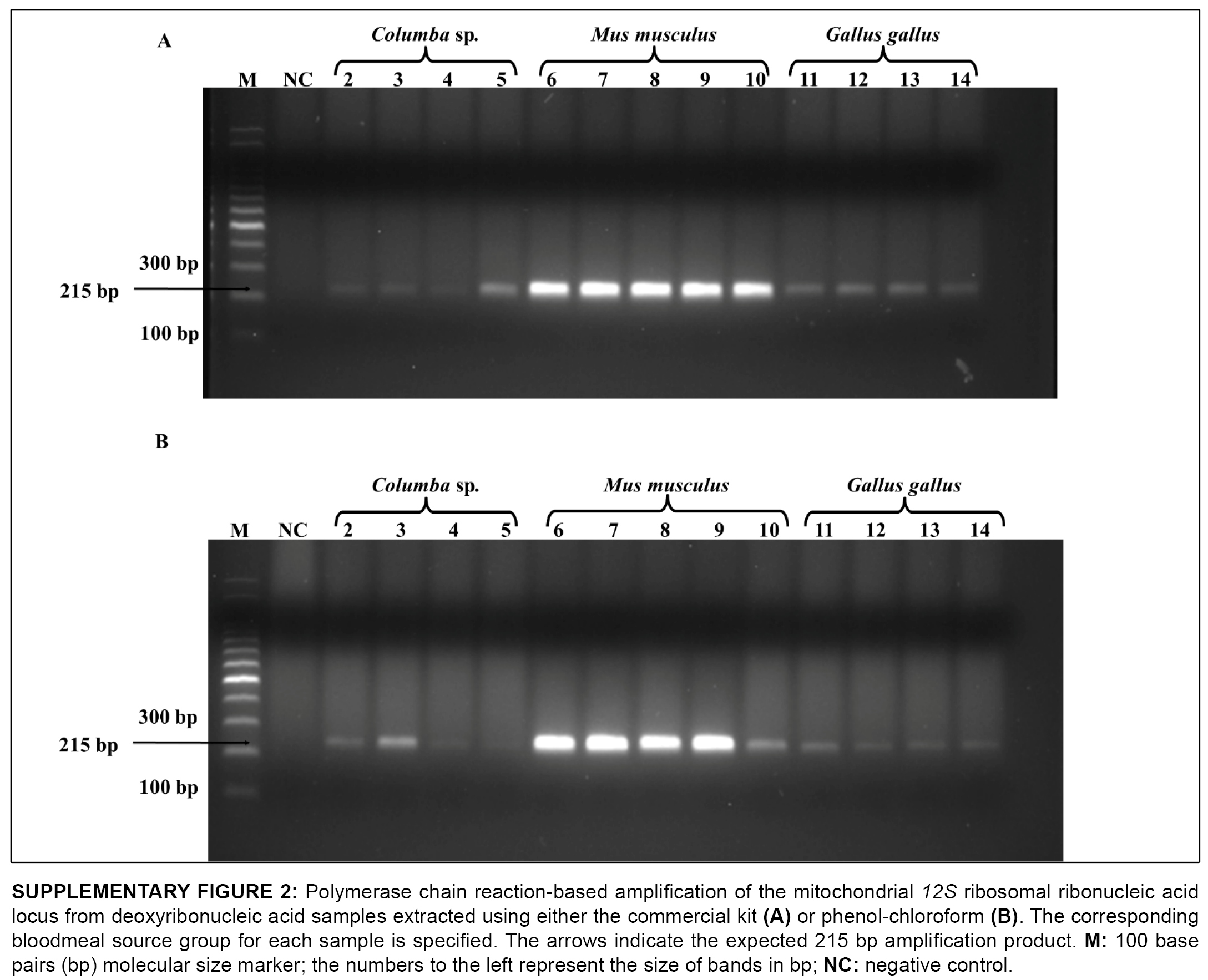

Supplement: Supplementary file 2 [file 1678-9849-rsbmt-53-e20200189-suppl2.jpg]
